# Targeted deep amplicon sequencing of *kelch 13* and *cytochrome b* in *Plasmodium falciparum* isolates from an endemic African country using the Malaria Resistance Surveillance (MaRS) protocol

**DOI:** 10.1186/s13071-020-4005-7

**Published:** 2020-03-14

**Authors:** Mariangela L’Episcopia, Julia Kelley, Dhruviben Patel, Sarah Schmedes, Shashidahar Ravishankar, Michela Menegon, Edvige Perrotti, Abduselam M. Nurahmed, Albadawi A. Talha, Bakri Y. Nour, Naomi Lucchi, Carlo Severini, Eldin Talundzic

**Affiliations:** 1 Department of Infectious Diseases, Istituto Superiore di Sanità, Rome, Italy; 2 Atlanta Research and Education Foundation, VAMC, Atlanta, Georgia USA; 3 Association of Public Health Laboratories, Silver Spring, MD USA; 4 School of Biology, Georgia Institute of Technology, Atlanta, Georgia USA; 5 Cansford Laboratories, Pentwyn Business Centre, Cardiff, UK; 6 Faculty of Medical Laboratory Science, University of Gezira, Gezira, Sudan; 7 Department of clinical laboratory Sciences, College of Applied Medical Sciences, Juof University, Sakaka, Saudi Arabia; 8 Blue Nile Research National Institute for Communicable Diseases, University of Gezira, Wad Medani, Sudan; 9 Centers for Disease Control and Prevention, CGH, DPDM, Atlanta, GA USA

**Keywords:** *Plasmodium falciparum*, Drug resistance, Next-generation sequencing, Molecular surveillance

## Abstract

**Background:**

Routine molecular surveillance for imported drug-resistant malaria parasites to the USA and European Union is an important public health activity. The obtained molecular data are used to help keep chemoprophylaxis and treatment guidelines up to date for persons traveling to malaria endemic countries. Recent advances in next-generation sequencing (NGS) technologies provide a new and effective way of tracking malaria drug-resistant parasites.

**Methods:**

As part of a technology transfer arrangement between the CDC Malaria Branch and the Istituto Superiore di Sanità (ISS), Rome, Italy, the recently described Malaria Resistance Surveillance (MaRS) protocol was used to genotype 148 *Plasmodium falciparum* isolates from Eritrea for kelch 13 (*k13*) and cytochrome *b* (*cytb*) genes, molecular markers associated with resistance to artemisinin (ART) and atovaquone/proguanil (AP), respectively.

**Results:**

Spanning the full-length *k13* gene, seven non-synonymous single nucleotide polymorphisms (SNPs) were found (K189N, K189T, E208K, D281V, E401Q, R622I and T535M), of which none have been associated with artemisinin resistance. No mutations were found in cytochrome *b*.

**Conclusion:**

All patients successfully genotyped carried parasites susceptible to ART and AP treatment. Future studies between CDC Malaria Branch and ISS are planned to expand the MaRS system, including data sharing, in an effort to maintain up to date treatment guidelines for travelers to malaria endemic countries. 
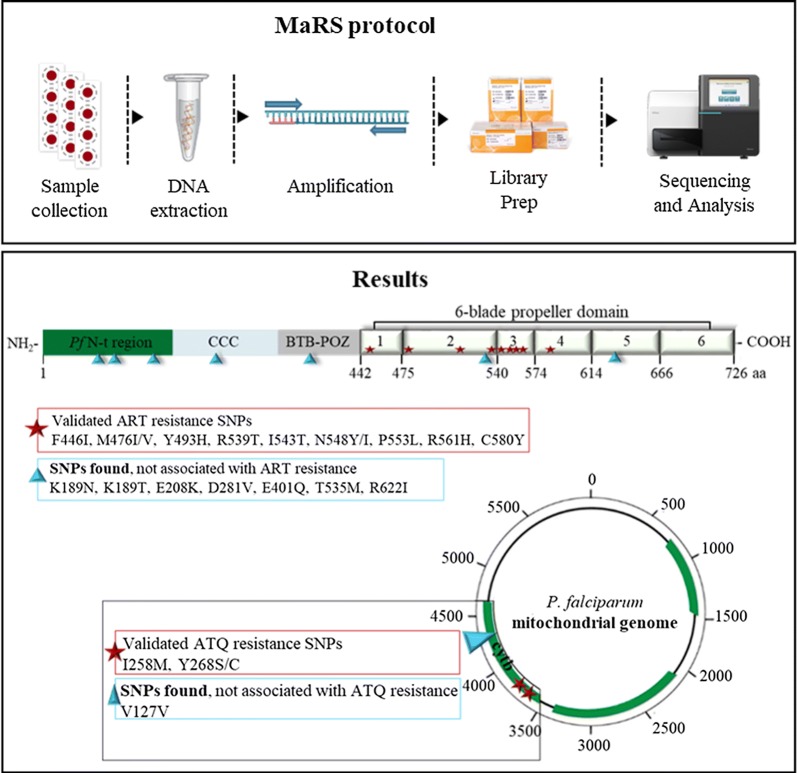

## Background

In the last decade, total malaria cases have been reduced by 40% worldwide [1, 2] leading to a dramatic reduction in mortality in children, especially in sub-Saharan Africa. This was achieved through the deployments of vector control measures, accurate diagnosis, and treatment of uncomplicated *Plasmodium falciparum* malaria with artemisinin-based combination therapies (ACTs). Despite this overall progress, thousands of travel-related malaria cases are imported into the European Union (EU) and the USA [3, 4]. The European Centre for Disease Prevention and Control (ECDC) estimate that an average of 8000 malaria cases are imported to the EU annually, most of which are travelers returning home after visiting friends and relatives in Africa [4]. In the USA, according to the Centers for Disease Control and Prevention (CDC), approximately 1700 malaria cases are imported annually to the country [3].

Both the EU and the USA, recommend the use of atovaquone/proguanil (AP) for chemoprophylaxis [5–7]. In the USA, AP is used as a primary treatment choice for imported uncomplicated *P. falciparum* cases, while in the EU, two ACTs, artemether-lumefantrine (AL) and dihydroartemisinin/piperaquine (DHA/PPQ) are licensed as treatment options for uncomplicated *P. falciparum* malaria [8–10]; AP can be used if an ACT is not available [10].

Single nucleotide polymorphisms (SNPs) in the cytochrome *b* (*cytb*) gene, in the mitochondrial genome of *P. falciparum*, confer resistance to AP. The putative I258M and Y268S/C SNPs have been associated with AP resistance [11, 12]. In comparison, resistance to artemisinin derivatives, given as part of ACTs, such as AL, are reported with parasites carrying SNPs in the kelch 13 (*k13*) gene. Specifically, the F446I, N458Y, M476I, Y493H, R539T, I543T, P553L, R561H and C580Y SNPs are confirmed to confer resistance to artemisinin. [13]. The *k13* gene provides a validated molecular marker to detect, track and monitor the emergence and spread of artemisinin-resistant SNPs [14–16]. While a number of other *k13* SNPs have been described in Africa, none have yet been confirmed to be associated with artemisinin resistance [17–19], except in a case described by Lu et al. [20] in which was shown that a *P. falciparum* imported into China from Equatorial Guinea was resistant to artemisinin *in vitro*.

Molecular surveillance of imported malaria cases is an important public health activity that can aid in the detection of drug-resistant *P. falciparum* parasites [21, 22]. Recent advancements in next-generation sequencing are providing a new way to rapidly detect and characterize drug-resistant malaria parasites, including minor parasite populations in mixed infection *P. falciparum* cases [23, 24]. These molecular data can be used to help keep chemoprophylaxis and treatment guidelines up to date for persons traveling to malaria endemic countries.

Towards this end, as part of a technology transfer training agreement between the CDC and the Istituto Superiore di Sanità, Rome, Italy, we used the recently published Malaria Resistance Surveillance (MaRS) [24] protocol to genotype SNPs in the kelch 13 and cytochrome *b* genes from 148 *P. falciparum*-positive samples from Eritrea.

## Methods

### Samples

A total of 148 *P. falciparum* dried blood spots (DBS) were used in this study; these were originally collected as part of a cross-sectional study between November 2013 and November 2014 in two regions (Barentu and Agordat) of Eritrea endemic for *falciparum* malaria, as previously described by Menegon et al. [25]. Samples were initially analyzed for *P. falciparum* infection using molecular diagnosis at the Gezira University (Wad Medani, Sudan) in a collaboration existing between Gezira University and the Eritrean Ministry of Health.

### DNA extraction

DNA was extracted using the QIAmp DNA Blood Mini Kit (Qiagen, Valencia, CA, USA) using the recommended guidelines in the original MaRS protocol [24].

### *Plasmodium falciparum* species confirmation and sample quality assurance

The previously described real-time PET-PCR method [26] was used to confirm *P. falciparum* infections and assess the quality of DNA from the filter blood spot samples. This method is based on self-quenching photo-induced electron transfer (PET) fluorogenic primers used in a multiplex real-time PCR to detect *Plasmodium* spp. and *P. falciparum*. Samples with a Cq value > 40 were considered *P. falciparum*-negative.

### Kelch 13 and cytochrome *b* PCR enrichment

The following primers were used: *k13* gene (forward: 5′-CTA TGA CGT ATG ATA GGG AAT CTG G-3′ and reverse: 5′-CTG GGA ACT AAT AAA GAT GGG CC-3′); cytochrome *b* gene (forward: 5′-CTA TTA ATT TAG TTA AAG CAC AC-3′ and reverse: 5′-ACA GAA TAA TCT CTA GCA CCA-3′). Previously described PCR conditions were used [24]. *Plasmodium falciparum kelch13* wild-type strains HB3, 7G8 and *P. falciparum kelch13* C580Y mutant strain MRA1236 (ATCC) were used as controls. DNA isolated from whole blood of individuals without malaria was used as a negative control.

### PCR product purification, normalization, pooling, library prep and sequencing

Briefly, PCR products were purified and normalized using the SequalPrep Kit (Cat # A1051001; Thermo Fisher Scientific, Waltham, USA). PCR products were pooled in 10.0 µl volumes for each sample. Library prep was performed using the Illumina Nextera XT kit (Cat # FC-131-1096 and FC‐131‐1002; Illumina, San Diego, USA). Pooled fragments were assessed for correct size using the Agilent D5000 ScreenTape Station (Cat # G2940C, # 5067-4626, and # G2940CA; Agilent Technologies, Santa Clara, USA) and DNA concentration checked using the Qubit 3.0 Flurometer (Cat #Q33216 and #Q32853; Life Technologies Corporation, Carlsbad, USA). Sequencing was performed using the MiSeq v2 reagents using the 500 cycle kit (Cat # MS-102-2003; Illumina MiSeq reagents v2, San Diego, USA).

### Data analysis

The next-generation sequencing analysis toolkit (NeST) (https://github.com/CDCgov/MaRS) was used to call non-synonymous and synonymous single nucleotide polymorphisms (SNPs) in *k13* and *cytb* genes, respectively. All SNPs were also confirmed using the Geneious Prime software (www.geneious.com).

### MaRS protocol details

Details about the laboratory and data analysis protocols can be found at https://github.com/CDCgov/MaRS. This protocol is based on collating and mapping SNPs associated with antimalarial drug resistance by using a targeted amplicon deep sequencing (TADS) approach.

## Results

### Sequencing outcome using dried blood spots

**Fig. 1:**
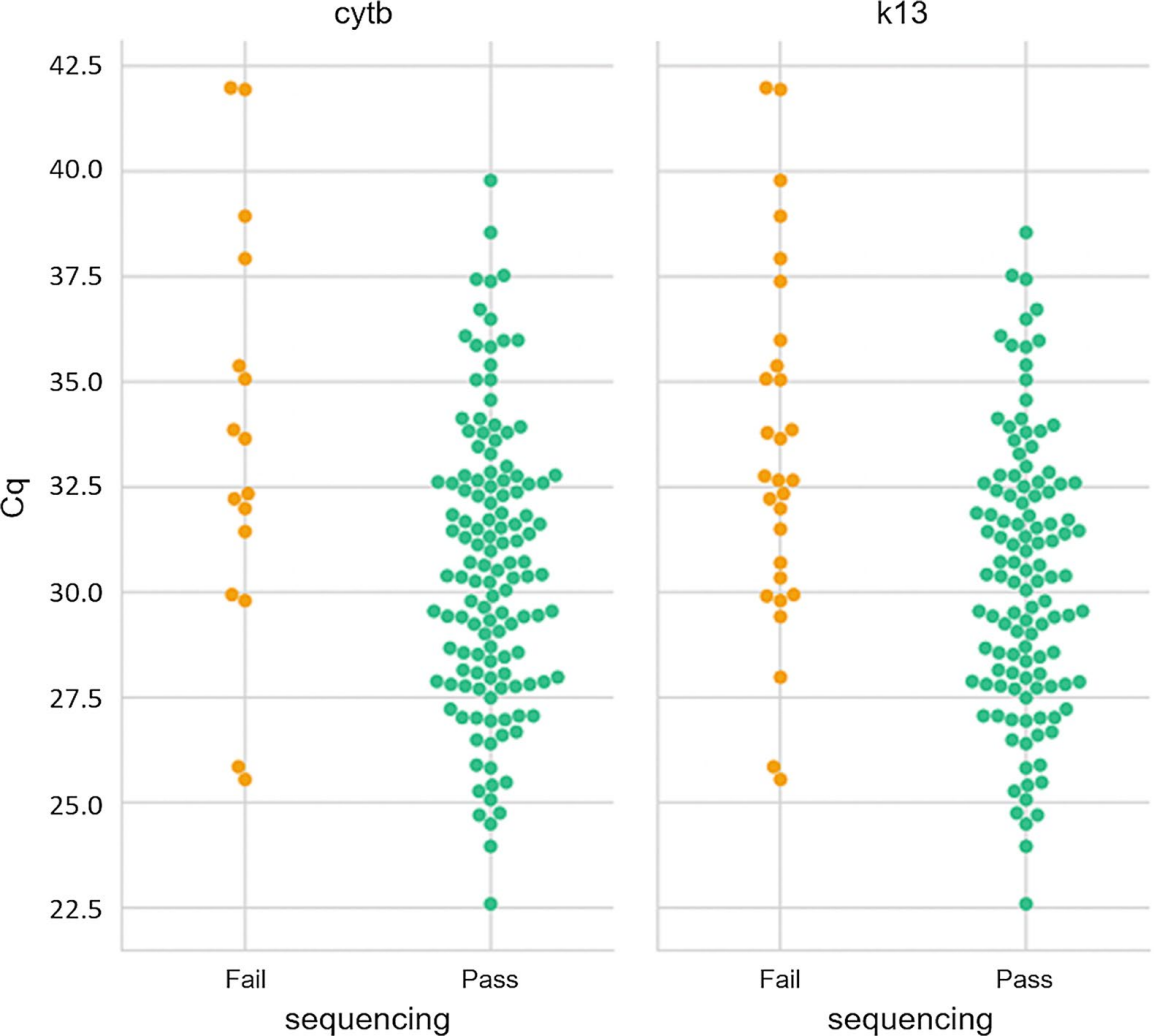
Sequencing outcome for kelch 13 and cytochrome *b* genes. Sequencing pass (green dots) or fail (red dots) is shown on the x-axis and Cq values for each sample on the y-axis. Successful sequencing data were obtained for 82.6% (119/144) and 91.7% (132/144) samples for the *k13* and *cytb* genes, respectively

PET-PCR was performed on all 148 samples in duplicate. A total of 144 out of 148 screened samples were positive for both the genus *Plasmodium* and *P. falciparum* based on the Cq values (mean of 30.9). Out of the positive samples, successful sequencing results were obtained for 82.6% (119/144) and 91.7% (132/144) samples for the *k13* and *cytb* genes, respectively, as shown in Fig. 1.

### Read depth for SNPs associated with malarial drug-resistant *k13* and *cytb*

**Fig. 2:**
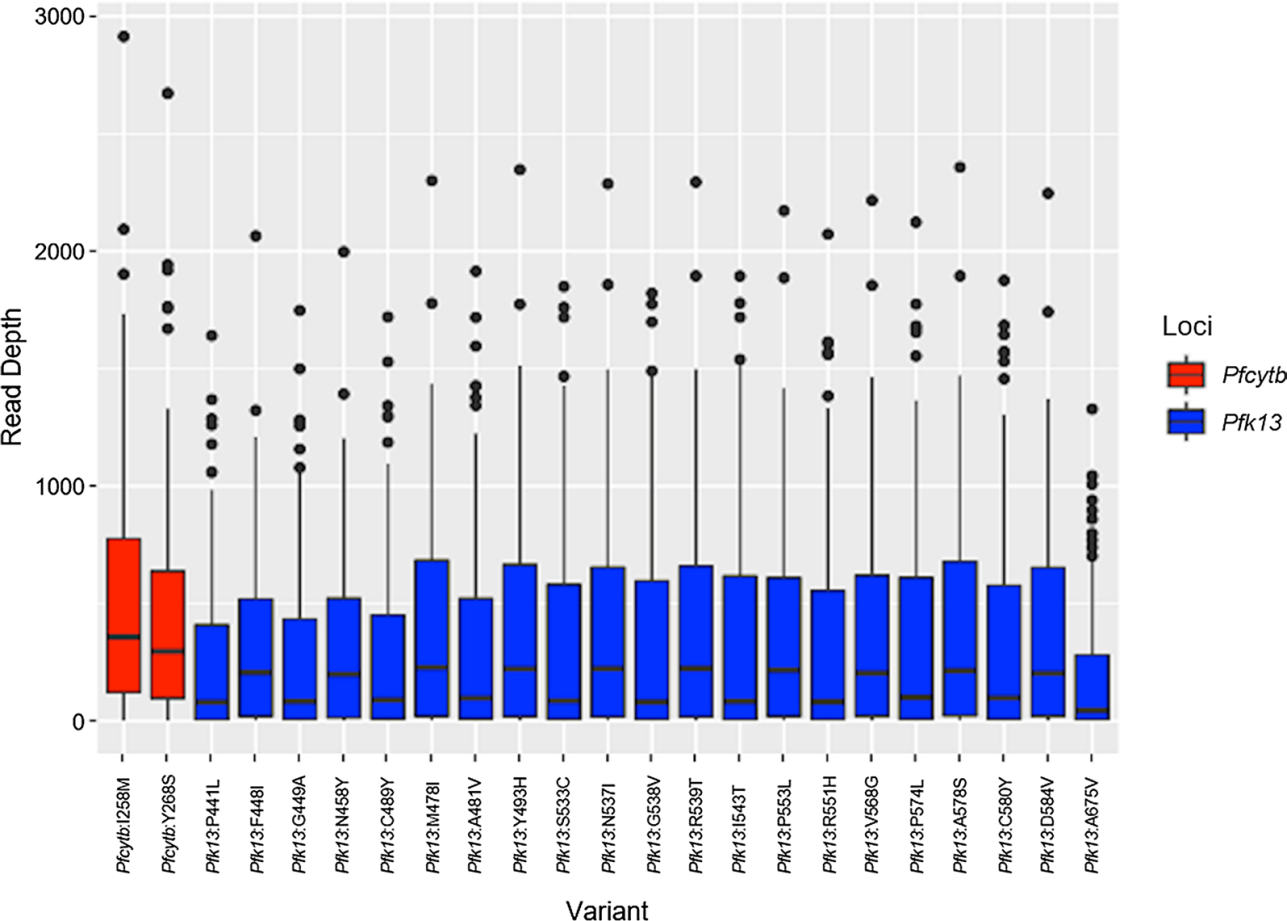
Read depth for SNPs associated with malarial drug resistance in the *Plasmodium falciparum cytb* and *k13*. Middle bar, median; upper box hinge, 75th percentile; lower box hinge, 25th percentile; upper whisker, largest value no further than 1.5 × IQR (inter-quartile range or distance from first and third quartiles) from the hinge; lower whisker, smallest value at most 1.5 × IQR from the hinge; dots, outlying samples

Read depth at 2 SNPs in the *cytb* and 21 SNPs (9 validated SNPs and 12 candidate SNPs based on the 2018 WHO artemisinin report) in *k13* for 119 and 132 samples, respectively, were analyzed (Fig. 2). Median read depth across SNPs in *cytb* was 310.5× coverage. The median read depth across SNPs in *k13* was 126X coverage.

### Kelch 13

Spanning the full-length *k13* gene in the 82.6% (119/144) successfully sequenced samples, seven non-synonymous SNPs were detected (Table 1). Of the seven non-synonymous mutations detected, five were found outside the propeller domain region: K189N, K189T, E208K, D281V and E401Q. Of these, the most frequent SNP was K189T found in 59.7% (71/119) of samples at an allele frequency of 100%, except for one sample in which the polymorphism was identified as minor allele at an allele frequency of 43.0%. Inside the *k13* propeller domain, two non-synonymous SNPs were identified: R622I and T535M. R622I was detected as a major allele at an allele frequency of 100% and T535M as a minor allele at an allele frequency of less of 50% (Table 1).

**Table 1:** Prevalence of polymorphisms in *kelch 13* and cytochrome *b* genes in the analyzed *falciparum* isolates. K13 protein consists of a *Plasmodium*-specific N-terminal region (*Pf* N-t region), coiled-coil-containing domain (CCC), a Broad-complex, Tramtrack, Bric-a-Brac/Poxvirus and Zincfinger domain (BTB/POZ) and a C-terminal Kelch-repeat propeller domain (KREP)

Target gene	Codon position	Domain location	Reference sequence		Mutant sequence		Type	Allele frequency (%)	*n*/*N*
			Amino acid	Nucleotide	Amino acid	Nucleotide			
*Pfk13*	189	*Pf* N-t region	K	AA**A**	N	AA**T**	NS	100	4/119
	189	*Pf* N-t region	K	A**A**A	T	A**C**A	NS	100	70/119
	189	*Pf* N-t region	K	A**A**A	T	A**C**A	NS	43	1/119
	208	*Pf* N-t region	E	**G**AA	K	**A**AA	NS	100	10/119
	281	CCC	D	G**A**T	V	G**T**T	NS	100	2/119
	401	BTB/POZ	E	**G**AG	Q	**C**AG	NS	100	4/119
	622	KREP	R	A**G**A	I	A**T**A	NS	100	1/119
	535	KREP	T	A**C**G	M	A**T**G	NS	20	1/119
*Pfcytb*	127	–	V	GTG	V	GTG	Syn	100	2/132

*Note*: The boldface highlights the nucleotide base change

*Abbreviations*: n, number of samples containing mutant allele; N, total number of successfully sequenced samples; NS, non-synonymous mutation

### Cytochrome *b*

A total of 91.7% (132/144) were successfully sequenced for *cytb*. Of these, 98.5% (130/132) carried the wild-type 258I and 268Y alleles. Two samples were identified with the V127V synonymous mutation (Table 1).

## Discussion

Active surveillance of imported malaria cases is an important public health activity in the USA and the EU [27, 28]: it plays a pivotal role in estimating the incidence in imported malaria cases, preventing malaria reintroduction, and providing chemoprophylaxis guidelines to travelers to malaria endemic regions. Historically, anti-malaria drug resistance genotyping was performed using traditional low throughput sequencing methodologies, such as Sanger sequencing [29]. However, currently, next-generation sequencing and accompanying standardized bioinformatics tools provide advanced and rapid protocols for monitoring molecular marker genes involved in *P. falciparum* drug resistance [24].

Based on the WHO guidelines, routine malaria surveillance is critical for the intervention in all malaria endemic and non-endemic countries, with the goal to reduce the incidence of malaria, eliminate the disease and prevent its re-establishment through detection and characterization of malaria parasites [30]. To achieve this goal, laboratory and analysis protocols related to malaria molecular surveillance activities need to be standardized, have appropriate quality assurance systems in place, and provide information in a timely fashion. The recent advances in next-generation sequencing (NGS), including high throughput and decreasing costs, meet these requirements and are making this technology more suitable for routine molecular surveillance of *P. falciparum* drug resistance genes in public health laboratories [24]. Notably, with this approach, lots of samples can be analyzed simultaneously, allow focusing on the full length of the genes, rather than only some regions. Toward this end, as part of a technology transfer arrangement between the CDC and the Istituto Superiore di Sanità, Rome, the recently developed Malaria Resistance Surveillance (MaRS) protocol was used to genotype *Pfk13* and *Pfcytb* genes using dried blood spot samples available for the present study. The *k13* and *cytb* genes were genotyped since AP, AL and DHA/PPQ are used for treatment of malaria cases imported into Italy.

Our results are in agreement with previously published reports on the *k13* gene from Africa, where a large number of non-artemisinin resistant associated SNPs have been reported. Notably, the K189T mutation, which is widely prevalent in African artemisinin-sensitive *P. falciparum* parasites [31] as well as in South East Asian parasites [32] was observed. We found no evidence of the putative I258M and Y268S/C SNPs associated with AP resistance [11, 12], suggesting that the use of AP for prophylaxis for travelers to Eritrea remains effective.

## Conclusions

The MaRS protocol used in this technology transfer training provides a standardized, high-throughput laboratory and analysis system for characterizing and tracking possible polymorphisms in molecular marker genes linked to drug resistance. The goal of this study was to evaluate the possibility to set up MaRS system also in Italy and to maintain active collaboration between the two institutes.

## Data Availability

The datasets generated during the present study are available in the PRJNA428490 repository, https://www.ncbi.nlm.nih.gov/Traces/study/?acc=SRP128900.

## Abbreviations

NGS: next-generation sequencing
CDC: Centers for Disease Control and Prevention
ISS: Istituto Superiore di Sanità
MaRS: Malaria Resistance Surveillance
*k13*: *kelch 13* gene
*cytb*: *cytochrome b* gene
ART: artemisinin
ATQ: atovaquone
AP: atovaquone/proguanil
SNPs: single nucleotide polymorphisms
ACT: artemisinin-based combination therapy
ECDC: European Centers for Disease Control and Prevention
AL: artemether/lumefantrine
DHA: dihydroartemisinin
PPQ: piperaquine
DBS: dried blood spot
PET: photo-induced electron transfer
PCR: polymerase chain reaction
NeST: next-generation sequencing analysis toolkit
IQR: inter-quartile range
